# Occlusal Features and Temporomandibular Joint Disorder: A Cross-Sectional Study

**DOI:** 10.1155/2024/8715166

**Published:** 2024-05-18

**Authors:** Mustafa M. Al-Khatieeb, Layth M. Nissan, Yasir R. Al-Labban, Mushriq Abid

**Affiliations:** Orthodontic Department, College of Dentistry, University of Baghdad, Baghdad, Iraq

## Abstract

**Background:**

Understanding the correlation between temporomandibular joint disorder (TMD) parameters and various occlusal features can play a significant role in diagnosing and treating occlusal problems and their potential consequences on TMD.

**Objectives:**

To investigate the relationship of occlusal features and some of the TMD parameters. *Study Design and Sampling*. The current cross-sectional study was conducted on 200 TMD patients seeking dental treatment at different dental centers in Baghdad city, aged 18–35 years. All participants underwent assessment for occlusal features, which were molar and canine classifications, overjet, posterior buccal or lingual crossbites, and overbites, and TMD parameters (muscle pain, TMJ pain, and clicking) using the Chi-square test for statistical analysis.

**Results:**

Regarding molar classification, there were strong positive correlations between subjects with TMD and having different molar classifications (class I, II, and III) bilaterally or unilaterally; furthermore, there were strong positive correlations between subjects with TMD and having different canine classifications. Moreover, there were significant correlations between subjects with increased, normal, or decreased overjet and the TMD parameters. In addition, there were weak positive correlations between TMD occurrence and buccal or lingual posterior crossbite. On the other hand, there were significant correlations between subjects with a decreased overbite and the presence of TMD parameters.

**Conclusion:**

TMD had a multifactorial background rather than dependability on a specific molar or canine classification type. There was also a correlation between overjet and TMD muscle pain, while decreased overbite was correlated to muscle and TMJ pain. Conversely, there is no vital correlation between posterior buccal or lingual crossbite occurrence and TMD parameters.

## 1. Introduction

The masticatory system, consisting of the jaws, muscles, temporomandibular joint, teeth, and the tissues that support them, is considered a functional system [1]. The vascular and nervous systems work in tandem with this system [2, 3]. In general, dental healthcare professionals and, to a lesser extent, orthodontists face the unpleasant problem that the most common reason for which patients try to find medical care before, during, or after orthodontic treatment is due to pain or malfunctioning [4]. The most typical pain in the orofacial region is odontogenic, although nonodontogenic orofacial pain can occur from a temporomandibular disorder, which is not uncommon [5].

The American Academy of Orofacial Pain (AAOP) defines temporomandibular disorders (TMD) as a general term encompassing several clinical complaints connected to the masticatory muscles, the temporomandibular joint (TMJ), and/or associated orofacial structures [6]. Therefore, TMD frequently includes pain, sounds from the joint itself, and dysfunction [7]. The most noticeable symptom is TMJ popping, which frequently causes a mandibular shift while moving, occasionally a smaller mouth opening, discomfort, and headaches, especially while eating [8].

The TMDs are believed to be one of the leading causes of pain in the craniofacial region, excluding pain from sources other than the teeth. This is because the most prevalent symptom is pain from the masticatory muscles, supporting soft tissues, and the TMJ, such as a dull, persistent ache overlying the temple muscles with sporadic radiation to other structures such as the head, neck, ear, and teeth [9, 10]. Other symptoms can also include a restricted opening, fatigue, stiffness, joint locking, clicking, and crepitus, representing the most consistent features associated with TMD [10–12]. The prevalence of TMD signs and symptoms is widely reported in different research [11–13]. TMD is stressed to be a multifactorial issue, so orthodontists have made significant studies regarding craniofacial orthopedics involving the TMJ. As a result, the role of orthodontics in improving or worsening TMJ disorder is currently being studied in various specialities other than orthodontics, for instance, oral medicine [6, 7, 14, 15], oral diagnosis [16], prosthodontics [17, 18], and forensic dentistry [19].

Malocclusion has been identified as a risk factor for TMD [10–16]. Solberg et al.[19] noted that more extensive TMJ changes may be related to more prolonged exposure to malocclusion. To the best of our knowledge, the potential association between TMD and occlusal features has never been investigated in Iraqi populations; therefore, the current study aimed to evaluate any possible relationship between occlusal features and some TMD criteria to provide better knowledge and understanding of the potential association between occlusal features and TMD. Consequently, the data obtained from the present study can be applied to improve methodological approaches. The null hypothesis was that there would be no relation between occlusal features and TMD parameters in TMD patients.

## 2. Methods

### 2.1. Study Design and Sampling

This is a cross-sectional observational study. The study protocol was reviewed and accepted by the ethical committee in the Orthodontic Department at the College of Dentistry, University of Baghdad (Project Code number: 804423). All the patients were made aware of the study process and encouraged to participate in the investigation.

Since it was decided to include consecutive TMD patients seeking dental treatment at different centers in Baghdad, the minimum number of necessary patients was 150, which was determined using previously reported studies [20, 21]. For improved results and dependability and to assure more statistical power, this was raised to 200 subjects. The sample was selected to involve an approximately equal number of females and males to exclude gender bias. All met the inclusion criteria and had the same age group (18–35). The sample collection was done between 2018 and 2022.

### 2.2. Inclusion and Exclusion Criteria

The inclusion criteria were the presence of one or more TMD symptoms (muscle pain, TMJ pain, and TMJ clicking), the presence of a complete set of permanent dentitions excluding third molars, the availability of evaluable molar and canine relationships, and incisor overjet and overbite.

The exclusion criteria were teeth extraction, no crowns and bridges or any prosthetic treatments, occlusal rehabilitations and adjustments, a recent history of trauma to the head and neck regions, history of TMJ surgery and/or orthodontic or orthognathic surgery treatment, current odontogenic pain compliance (as this could overlap or mask TMD pain), and previously diagnosed and treated TMD problem; as such, patients are likely to have received some treatment such as medicines that might mask TMD symptoms (for example, nonsteroidal anti-inflammatory drugs, anticonvulsants, tricyclic antidepressants, and muscle relaxants), patients with disabilities, dental and/or facial deformities, no history of growth or developmental disorders, no rheumatic disease (e.g., arthritis), or no infection or neoplasms in the jaws and associated structures.

### 2.3. Measurement Instrument

Occlusal features: All participants underwent assessment for occlusal features based on a previous protocol [19, 22], classified after clinical examination according to their specific occlusal features.

According to molars (Angle's) classification [22]: class I, II, or III (bilateral evaluable class) and the presence of class I and class II, class I and III, or class II and III on the two opposite sides.

According to canine classification [23]: class I, II, or III (bilateral evaluable class), presence of class I and Class II, class I and III, or class II and III on the two opposite sides.

According to overjet [21, 24]: class I (normal overjet of 1–3 mm), class II (increased overjet of more than 3 mm), or class III (decreased overjet of less than 1 mm).

According to posterior crossbite availability [25–27]: bilateral crossbites (with or without a shift in the midline), unilateral scissor bites (with or without a shift in the midline), or bilateral scissor bites (with or without a shift in the midline).

According to overbite [21, 24]: class I (normal overbite of 1–4 mm), class II (increased overbite of more than 4 mm), or class III (decreased overbite of 1 mm and less).

The above occlusal features were grouped separately for the availability of molars, canines, overjet, overbite, and posterior crossbites.

Temporomandibular disorders (TMD) parameters: It was assessed based on the diagnostic features guidelines for temporomandibular disorders [22, 28] by two expert examiners, but the assessment concentrated primarily on diagnosing the existence of joint pain, clicking, and muscle pain. The muscle pain evaluation included unilateral and bilateral palpation of masticatory muscles by the index and third fingers with a standardized pressure of 2 lbs based on the passive muscle state [22, 23, 25]. The site of maximum tenderness may vary from subject to subject, and it is localized, so it is important to palpate in multiple regions in the specified area to determine the existence of tenderness (present or absent). The diagnostic evaluation showed the presence (unilaterally or bilaterally) or absence of joint pain by palpation of TMJ sites by the index and the third fingers by a standardized pressure of 1 lb in the joint area [22, 23, 25]. At last, TMJ clicking was evaluated to determine whether there was a presence (unilaterally or bilaterally) or absence of popping on the existing site by palpation [2, 22, 23, 25, 29].

### 2.4. Statistical Analyses

A statistical analysis was performed using the IBM SPSS software platform version 20 (IBM, Chicago, USA). Number and percentage were used for descriptive statistics, while Chi-square correlation analysis was used for inferential statistics. *R*-value greater than 0.7 was considered a supportive, strong positive correlation [25], and the following level of significance was considered: high significance at *P* ≤ 0.001, significance at *P* < 0.05, and nonsignificance at *P* > 0.05. All participants were assessed by the same two trained examiners, and interexaminer reliability was confirmed statistically before starting the study project.

## 3. Results

Interexaminer reliability for occlusal features and TMD parameters exhibited excellent agreement (<0.75).

In regard of Angle's classification, within the whole sample of 200 subjects, there were relatively high number of subjects with an absence of muscle pain on palpation (*N* = 51 for class I, *N* = 22 for class II, and *N* = 16 for class III) and absence of TMJ pain parameters on palpation (*N* = 54 for class I, *N* = 27 for class II, and *N* = 24 for class III) and relatively high number of subjects with a presence of TMJ clicking parameter on palpation (*N* = 46 for class I, *N* = 29 for class II, and *N* = 22 for class III) for symmetrical Angle's classification (bilateral Angle's class I, II, and III), while for asymmetrical Angle's classification (having Angle's class I versus class II and class III and Angle's class II versus class III), there were relatively high number of subjects with absence of muscle pain on palpation (*N* = 20 for class I versus class II, *N* = 9 for class I versus class III, and *N* = 4 for class II versus class III) and absence of TMJ pain parameters on palpation (*N* = 20 for class I versus class II, *N* = 10 for class I versus class III, and *N* = 2 for class II versus class III) and relatively high number of subjects with presence of TMJ clicking parameter on palpation (*N* = 20 for class I versus class II, *N* = 9 for class I versus class III, and *N* = 3 for class II versus class III). When the Angle's classification was symmetrical (bilateral Angle's class I, II, or III), there were highly significant correlations (*P* ≤ 0.001) between bilateral Angle's classification (class I, II, and III) and TMD because of the presence of TMJ clicking and absence of muscle or TMJ pain. In contrast, if the Angle's classification was asymmetrical on both sides, there was no significant correlation (*P* > 0.05) between them. On the other hand, there were strong positive correlations (*R* > 0.7) between subjects with TMD (absence of muscle and TMJ pain and the presence of it and the presence of TMJ clicking and the absence of it) and people having different Angle's classifications (class I, II, and III) bilaterally or unilaterally (Table 1).

In regard to canine classification, there were relatively high number of subjects with an absence of muscle pain on palpation (*N* = 43 for class I, *N* = 18 for class II, and *N* = 21 for class III) and absence of TMJ pain on palpation (*N* = 59 for class I, *N* = 19 for class II, and *N* = 25 for class III), while there was also a relatively high number of subjects with a presence of TMJ clicking on palpation for symmetrical canine classification (*N* = 52 for class I, *N* = 25 for class II, and *N* = 26 for class III) and asymmetrical classifications (*N* = 15 for class I versus class II, *N* = 7 for class I versus class III, and *N* = 5 for class II versus class III); in addition, there were strong positive correlations (*R* > 0.7) between subjects with TMD (absence of muscle and TMJ pain and the presence of it and the presence of TMJ clicking and the absence of it) in people having different canine classifications (class I, II, and III) symmetrical or asymmetrical; none the less, there were high significant correlations (*P* ≤ 0.001) between subjects with the presence of TMJ clicking and absence of muscle and TMJ pain and having symmetrical canine class I and class III; moreover, there was a significant correlations (*P* < 0.05) between subjects with the presence of TMJ clicking and absence of muscle and TMJ pain in symmetrical class II canine classification; furthermore, no significant correlations (*P* > 0.05) were emphasized between subjects with presence of TMD (muscle and TMJ pain and clicking) and having one side canine class I, and the other sides were canine class II or class III, and the same thing happened when one side was canine class II and the other side was class III (Table 2).

Concerning the correlation between TMD parameters and overjet for the total sample, 110 subjects (55%) exhibited increased overjet (more than 3 mm) correlated with muscle pain, 67 subjects (34%) had normal overjet, and 23 subjects (12%) had decreased overjet correlated with muscle pain, while regarding TMJ pain and clicking correlated with overjet, the highest percentage of subjects showed normal overjet, while the intermediate percentage and low percentage exhibited increased and decreased overjet, respectively. Additionally, there were highly significant correlations (*P* ≤ 0.001) between the amount of overjet (whether normal, increased, or decreased) and the occurrence of TMD parameters (muscle and TMJ pain and clicking) (Table 3).

About horizontal relation (buccal or lingual posterior crossbite), whether unilateral or bilateral occurrence with the presence or absence of TMD (muscle pain, TMJ pain, and TMJ clicking), all the percentage values were low and close together; into the bargain, there were weak positive correlations (*R* < 0.3) between TMD occurrence and buccal or lingual posterior crossbite; furthermore, there was a nonsignificant correlation (*P* > 0.05) between the presence of TMD problems and the occurrence of posterior buccal unilateral crossbite whether with or without shift in the midline (Table 4).

Regarding the correlation between TMD parameters and overbite for the total sample, 53 subjects (27%) with muscle pain showed an increased overbite (more than 4 mm), 112 subjects (56%) with muscle pain had a normal overbite, and 35 subjects (18%) had a decreased overbite. Regarding the subjects with TMJ pain and clicking, the highest percentage showed normal overbites, and at the same time, intermediate percentages and low percentages exhibited increased and decreased overbites, respectively. In addition, there were highly significant correlations (*P* ≤ 0.001) between subjects with a decreased overbite and the presence of TMD parameters (muscle pain, TMJ pain, and clicking). Furthermore, there was a significant correlation (*P* < 0.05) between normal overbite and TMD parameters. On the other hand, there was a nonsignificant correlation (*P* > 0.05) between subjects with an increased overbite and TMJ parameters (Table 5).

## 4. Discussion

Over the past few decades, ad hoc evidence has gradually diminished the significance of dental occlusion as the primary risk factor for temporomandibular joint and jaw muscle problems [24]. However, multiple variable models have frequently been used in the literature to analyze problems in dental occlusion and TMD [30–32], and it appears that the data obtained from studies using sophisticated statistical techniques is complex for general practitioners to handle and understand. Consequently, it was proposed that descriptive and straightforward statistical research could be even more beneficial in providing readers with takeaway information on the subject [33]. Asymmetry of dental occlusion, defined as not having the same molar Angle and canine classification on both sides, has long been recognized in orthodontics as a condition that needs to be treated [34–37]. However, its actual relationship to TMD has been determined to a lesser extent.

This investigation's straightforward design was based on the idea that asymmetry in tooth occlusion between the two sides (that is, with varying molar Angle and canine classes and buccal or lingual crossbites “with or without shift”), overjet, and overbite was correlated or not with TMD. Overall, the results disprove the research concept, except for a particular condition with an asymmetric molar angle and canine classes, buccal or lingual crossbites “with or without shift”, overjet, and overbite, and the TMD.

In summary, the number of subjects with symmetrical molar Angle's classification (same on the left and right sides) was more likely to have TMJ clicking without muscle and TMJ pain in different classes (I, II, or III), and it was not the same as that of asymmetric classes. Nevertheless, there are matchings in manner with the conceivable combinations of asymmetrical Angle classes on the left and right sides (i.e., class I versus class II, class I versus class III, and class II versus class III), even though there were highly significant correlations between the bilateral Angle's classification and the TMD because of the presence of TMJ clicking and absence of muscle or TMJ pain. In contrast, if the Angle's classification was asymmetrical on both sides, it had no significant correlation between them. Furthermore, there were robust positive correlations between subjects with TMD (absence of muscle and TMJ pain and the presence of it and the presence of TMJ clicking and the absence of it) and people having different Angle's classifications bilaterally or unilaterally. The present study reported that subjects with malocclusion with different Angle classifications were not considered at risk of TMD. The results came in line with Sabah [16] and Aboalnaga et al. [21] but disagreed with Sujatha et al. [2]. This controversy in the literature can be due to the muscular endurance and psychological health differences [38, 39], which were at least as apparent as those between different ethnic subjects with TMD and malocclusion; furthermore, the methodology differences in depending variable analysis, the absence of attempts to prejudice between dental- and skeletal-based asymmetries, and overlapping between TMD parameters can all affect the results.

With regard to canine classification, it can be inferred that the resulting pattern in the number of subjects with TMD parameters and canine classification was analogous to that of molar Angle classification; both of them showed highly significant correlations between TMD parameters and symmetrical molar or canine classifications and a nonsignificance correlation between the TMD parameters and asymmetrical molar or canine classifications. These findings for bilateral canine class I, II, or III agree with previous studies [21, 40]. Despite these interesting results, future research must examine the significance and clinical applicability of this finding.

Concerning the overjet, there were highly significant correlations between the number of subjects with an increased, normal, or decreased overjet and the TMD parameters due to the relatively high number of subjects with muscle pain in palpation with an increased overjet; thus, this result was not too deviated from the literature [23, 41–43], while the current study disagreed with the findings of others [21, 44]. In a nutshell, there was a correlation between subjects with an increased overjet and TMD problems (muscle pain).

It can be assumed that posterior buccal or lingual crossbite occurrence probably has no role in TMD parameters. This result agreed with Aboalnaga et al. [21], who stated that static occlusal variables had no significant effect on TMD problems. In addition, Manfredini et al. [29] formerly searched and found that interferences in the posterior segments could not anticipate TMJ problems. In general, orthodontists should concentrate on creating a balanced and stable occlusion rather than creating a particular type of occlusion, taking into account that the obvious posterior segment instability that is detected in children should be treated orthopedically as early as possible, especially if it is associated with TMD problems, to prevent future complications of the TMJ complex system.

There was highly significant correlation between subjects with decreased overbite and the presence of TMD parameters; this result can agree with other studies [41, 45, 46], which reported a significant relation between TMD problems and increased vertical dimension, or decreased overbite was associated with a higher prevalence of TMD symptoms (i.e., muscle pain); this might be because of subjects with a decreased overbite, which allows the mandible to move more freely and can cause joint overloading and eventually trigger the onset of TMD than those with an increased overbite (deepbites), while other studies [39, 42, 43] were at loggerheads with the current study, which reported the relationship between TMD muscle pain and decrease facial height (increased overbite); on the other hand, there was a nonsignificant correlation between subjects with increased overbite and TMJ parameters because there were close percentage values of TMJ parameters; this agreed with John et al.[44]. In a brief statement, a decreased overbite was correlated to muscle and TMJ pain.

Overall, it is essential to point out that this study offers information that may be utilized in the near future to improve methodological strategies. This study should be interpreted cautiously, but it is possible to argue that orthodontic indications for correcting occlusal problems should not be justified with the need to treat or prevent disorders of the jaw muscles and associated TMJ due to the nonsignificant if any, correlation with TMD. This is especially crucial given the amount of research that concentrates on potential treatments for malocclusion rather than their causes [47].

## 5. Clinical Considerations and Conclusions

1. The extemporaneous molar Angle and canine relations would not be assumed as clinical prognosticators for TMD because of the superimposition between the variables.
2. There appears to be a correlation between increased overjet and TMD muscle pain. In contrast, decreased overbite can be correlated with muscle and TMJ pain. Conversely, posterior buccal or lingual crossbite occurrence and TMD parameters do not play an essential role.
3. It is recommended that future research on the subject take a cautious approach to assessing the indications for orthodontic treatment that are related to TMJ pathology.

## Figures and Tables

**Table 1:** The number, percentage, Chi-square, and level of significance of TMD parameters in different Angle's classification.

Angle's class	Muscle pain		TMJ pain		Clicking		Chi-square	*P* value (significance)
	Present (*N* = 78)	Absent (*N* = 122)	Present (*N* = 63)	Absent (*N* = 137)	Present (*N* = 129)	Absent (*N* = 71)		
I	30 (39%)	51 (42%)	27 (43%)	54 (40%)	46 (36%)	31 (44%)	13.059	*P* ≤ 0.001 (HS)
II	12 (15%)	22 (18%)	12 (19%)	27 (20%)	29 (23%)	9 (13%)	19.05	*P* ≤ 0.001 (HS)
III	15 (19%)	16 (13%)	5 (8%)	24 (18%)	22 (17%)	8 (12%)	18.697	*P* ≤ 0.001 (HS)
I– II	12 (15%)	20 (16%)	12 (19%)	20 (15%)	20 (16%)	13 (18%)	4.69	0.170 (NS)
I–III	5 (6%)	9 (7%)	3 (5%)	10 (7%)	9 (7%)	5 (7%)	5.006	0.192 (NS)
II–III	4 (5%)	4 (3%)	4 (6%)	2 (2%)	3 (2%)	5 (7%)	1.167	0.811 (NS)
Correlation	0.969		0.921		0.876		—	
Significance	^*∗*^		^*∗*^		^*∗*^			

^*∗*^Strong positive correlation (*R* > 0.7); HS, highly significance at *P* ≤ 0.001; NS, nonsignificance at *P* > 0.05.

**Table 2:** The number, percentage, Chi-square, and level of significance of TMD parameters in different canine classifications.

Canine class	Muscle pain		TMJ pain		Clicking		Chi-square	Significance
	Present (*N* = 78)	Absent (*N* = 122)	Present (*N* = 62)	Absent (*N* = 138)	Present (*N* = 130)	Absent (*N* = 70)		
I	35 (45%)	43 (35%)	25 (40%)	59 (43%)	52 (40%)	30 (43%)	18.973	*P* ≤ 0.001(HS)
II	16 (21%)	18 (15%)	14 (23%)	19 (14%)	25 (19%)	8 (11%)	8.72	0.013 (S)
III	13 (17%)	21 (17%)	8 (13%)	25 (18%)	26 (20%)	7 (10%)	21.296	*P* ≤ 0.001 (HS)
I–II	7 (9%)	17 (14%)	8 (13%)	15 (11%)	15 (12%)	10 (14%)	5.448	0.070 (NS)
I–III	3 (4%)	16 (13%)	3 (5%)	15 (11%)	7 (5%)	11 (16%)	3.453	0.178 (NS)
II–III	4 (5%)	5 (4%)	4 (6%)	5 (4%)	5 (4%)	4 (6%)	0.155	0.926 (NS)
Correlation	0.926		0.911		0.837		—	
Significance	^*∗*^		^*∗*^		^*∗*^			

^*∗*^Strong positive correlation (*R* > 0.7); HS, highly significance at *P* ≤ 0.001; S, significance at *P* < 0.05; NS, nonsignificance at *P* > 0.05.

**Table 3:** The number, percentage, Chi-square, and level of significance of TMD parameters in different overjets.

Overjet	Muscle pain (*N* = 200)	TMJ pain	Clicking	Chi-square	Significance
Normal	67 (34%	110 (55%)	118 (59%)	15.3571	*P* ≤ 0.001 (HS)
Decreased	23 (12%)	5 (3%	29 (15%)	16.421	*P* ≤ 0.001 (HS)
Increased	110 (55%)	85 (43%)	53 (27%)	19.674	*P* ≤ 0.001 (HS)

HS, highly significance at *P* ≤ 0.001

**Table 4:** The number, percentage, Chi-square, and level of significance of TMD parameter occurrence in different posterior crossbites.

Posterior crossbite	Muscle pain		TMJ pain		Clicking		Chi-square	Significance
	Present (*N* = 8)	Absent (*N* = 10)	Present (*N* = 12)	Absent (*N* = 6)	Present (*N* = 13)	Absent (*N* = 5)		
Buccal posterior crossbite (BPCB) *N* = 18								
Unilateral (*N* = 12)								
With shift (*N* = 8)	2 (25%)	6 (60%)	5 (42%)	3 (50%)	5 (39%)	3 (60%)	3	0.223 (NS)
Without shift (*N* = 4)	0 (0%	4 (40%)	0 (0%)	4 (67%)	1 (8%)	3 (60%)	0.223 (NS)	0.336 (NS)
Bilateral (*N* = 6)								
With shift (*N* = 1)	1 (13%)	0 (0%)	1 (8%)	0 (0%)	1 (8%)	0 (0%)	N/A	N/A
Without shift (*N* = 5)	0 (0%)	5 (50%)	0 (0%)	5 (83%)	0 (0%)	5 (100%)	N/A	N/A
Lingual posterior crossbite (LPCB) (*N* = 4)								
Unilateral (*N* = 1)								
With shift (*N* = 0)	0 (0%)	0 (0%)	0 (0%)	0 (0%)	0 (0%)	0 (0%)	N/A	N/A
Without shift (*N* = 1)	1 (13%)	0 (0%)	1 (8%)	0 (0%)	1(8%)	0 (0%)	N/A	N/A
Bilateral (N = 3)								
With shift (*N* = 0)	0 (0%)	0 (0%)	0 (0%)	0 (0%)	0 (0%)	0 (0%)	N/A	N/A
Without shift (*N* = 3)	1 (13%)	2 (20%)	1 (8%)	2 (33%)	1(8%)	2 (40%)	N/A	N/A
Correlation	0.255		0.123		0.243			
Significance	†		†		†	

NS, nonsignificance at *P* > 0.05. ^†^weak positive correlation (*R* < 0.3). N/A, nonapplicable due to small sample size.

**Table 5:** The Number, percentage, Chi-square, and level of significance of TMD parameter occurrence in different overbites.

Overbite	Muscle pain	TMJ pain	Clicking	Chi-square	Significance
Normal	112 (56%)	134 (67%)	172 (86%)	13.259	0.020 (S)
Decreased	35 (18%)	18 (9%)	0 (0%)	34.055	*P* ≤ 0.001 (HS)
Increased	53 (27%)	48 (24%)	28 (14%)	8.725	0.070 (NS)

HS, highly significance at *P* ≤ 0.001. S, significance at *P* < 0.05. NS, nonsignificance at *P* > 0.05.

## Data Availability

The authors will provide the raw data without excessive delay to substantiate the conclusions of this article.

## Additional Points

*Limitations of the Study*. No solid experience can be drawn from a specific TMJ problem because TMD symptoms overlap and are multifactorial in their origin. There is an absence of attempts to distinguish between skeletal- and dental-based asymmetries, together with the lack of details on any pertinent related factors with TMD (such as psychological evaluation). The cross-sectional study design would preclude any etiological correlation between TMD and occlusal features from being established.
